# Towards Validation of a New Computerised Test of Goal Neglect: Preliminary Evidence from Clinical and Neuroimaging Pilot Studies

**DOI:** 10.1371/journal.pone.0148127

**Published:** 2016-01-29

**Authors:** Breda Cullen, David Brennan, Tom Manly, Jonathan J. Evans

**Affiliations:** 1 Mental Health and Wellbeing, Institute of Health and Wellbeing, University of Glasgow, Glasgow, United Kingdom; 2 Department of Clinical Physics, Institute of Neurological Sciences, Queen Elizabeth University Hospital, Glasgow, United Kingdom; 3 Medical Research Council Cognition and Brain Sciences Unit, Cambridge, United Kingdom; University College London, UNITED KINGDOM

## Abstract

**Objective:**

Goal neglect is a significant problem following brain injury, and is a target for rehabilitation. It is not yet known how neural activation might change to reflect rehabilitation gains. We developed a computerised multiple elements test (CMET), suitable for use in neuroimaging paradigms.

**Design:**

Pilot correlational study and event-related fMRI study.

**Methods:**

In Study 1, 18 adults with acquired brain injury were assessed using the CMET, other tests of goal neglect (Hotel Test; Modified Six Elements Test) and tests of reasoning. In Study 2, 12 healthy adults underwent fMRI, during which the CMET was administered under two conditions: self-generated switching and experimenter-prompted switching.

**Results:**

Among the clinical sample, CMET performance was positively correlated with both the Hotel Test (*r* = 0.675, p = 0.003) and the Modified Six Elements Test (*r* = 0.568, p = 0.014), but not with other clinical or demographic measures. In the healthy sample, fMRI demonstrated significant activation in rostro-lateral prefrontal cortex in the self-generated condition compared with the prompted condition (peak 40, 44, 4; *Z*_E_ = 4.25, p_(FWEcorr)_ = 0.026).

**Conclusions:**

These pilot studies provide preliminary evidence towards the validation of the CMET as a measure of goal neglect. Future studies will aim to further establish its psychometric properties, and determine optimum pre- and post-rehabilitation fMRI paradigms.

## Introduction

Goal management refers to the ability to keep in mind and work towards future goals, whilst simultaneously dealing with competing demands of other ongoing tasks. Goal management problems—termed goal neglect—are a common consequence of brain injury and neurological disorders, and can lead to significant disability and dependence on others. Goal neglect has been defined by Duncan and colleagues [1] as ‘disregard of a task requirement even though it has been understood and remembered. Subjectively it is as though the neglected requirement “slips the subject’s mind.”‘ (p. 257). In everyday life, goal neglect manifests as impaired ability to manage the multiple demands of instrumental tasks, such as cooking a meal. The functional consequences of this type of impairment can be devastating, even in the presence of no or minimal impairment of other cognitive domains or of general intellectual ability [2]. Tests that aim to emulate everyday goal management demands include the Six Elements Test and the Multiple Errands Test devised by Shallice and Burgess [2], the Modified Six Elements Test included in the Behavioural Assessment of the Dysexecutive Syndrome battery (BADS) [3], and the Hotel Test [4]. All of these tasks require the participant to switch between various sub-activities, while keeping overall rules and goals in mind, within a specified time limit. Impaired performance typically includes errors such as failure to carry out all required sub-tasks, and/or spending disproportionate time on some sub-tasks, despite knowing the overall goal.

Goal management is considered to be an executive function, and is closely related to other executive abilities such as multi-tasking and prospective memory (realisation of delayed intentions). There is now substantial evidence that rostral prefrontal cortex (PFC), approximating Brodmann Area 10 (BA10), is crucial to these types of abilities. The key functions of rostral PFC are thought to include aspects of working memory and episodic memory retrieval, mentalising, and multi-tasking [5]. Koechlin and Hyafil [6] proposed that rostral PFC ‘specifically subserves the ability to contingently switch back and forth between independent tasks by maintaining distractor-resistant representations of postponed tasks during the performance of another’ (p. 595).

Burgess and colleagues [7,8] put forward a theoretical explanation of rostral PFC function, which they termed the ‘gateway hypothesis’. Central to this hypothesis is the assertion that rostral PFC supports mechanisms that enable humans to attend to either environmental stimuli or to internally self-generated/maintained representations, as required. The gateway hypothesis can be expressed within the framework of Shallice and Burgess’s Supervisory Attentional System model of executive function [9]. It posits that there is, normally, continuous competition for activation of central representations between various sources, and that the gateway system ‘effects (through influence of attending behaviour) the coordination of stimulus-independent and stimulus-oriented cognition, specifically in situations where selection by this competition fails or is producing maladaptive behaviour’ [7] (p. 291).

More specifically, evidence from a range of lesion-based and functional neuroimaging studies supports the conclusion that rostro*medial* PFC supports processing relating to stimulus-oriented attending gain, and rostro*lateral* PFC facilitates switching to, maintaining, and voluntarily switching away from stimulus-independent cognition. Rostromedial PFC has been implicated in experimental conditions involving attending/responding to external stimuli and cues, whereas rostrolateral PFC shows greater activation during conditions involving internally self-generated or maintained representations, switching between internally- and externally-oriented attending, internal time estimation, and source memory retrieval (see [10] for a review). Much of the evidence in this area comes from working memory and prospective memory paradigms, however, which are generally highly structured and focused upon detection of and immediate response to designated prospective cues. Although, as noted above, rostral PFC has been linked with the construct of goal management, functional neuroimaging studies in this area have not employed task paradigms with demands that are closely akin to those of the various multi-element tests used in the clinical setting to detect goal neglect. There is a need to develop and validate such tasks in order to allow further investigation of the role of rostral PFC in goal management specifically.

There is also a need for suitable tasks which would allow assessment of goal neglect in functional neuroimaging studies before and after rehabilitation. Strategies such as Goal Management Training [11], external alerting [4], or modifications/combinations thereof [12] have been shown to change the behaviour of patients with goal neglect, but it is not yet known how underlying neural activation may change to reflect this. Functional neuroimaging studies of the effects of neurorehabilitation predominantly focus on motor and language function (see [13] for a review), and in those studies that do focus on goal-related behaviour, task paradigms typically use classic executive or attention tests rather than goal management tasks per se (e.g. [14,15]).

There are methodological challenges in conducting functional neuroimaging studies in this area, in addition to the lack of availability of goal neglect tests which are suitable for use in the scanning environment. The existing multi-element tests described above require writing, speaking and physical actions either in the context of a table-top set-up, or in a real life location such as a shopping precinct. These are clearly not compatible with a neuroimaging environment. A scanner-friendly test of goal neglect would need to be computerised and relatively brief, with minimal motor and speech demands, and relatively frequent behavioural events (e.g. task switches) to allow event-related statistical analysis.

**Fig 1:**
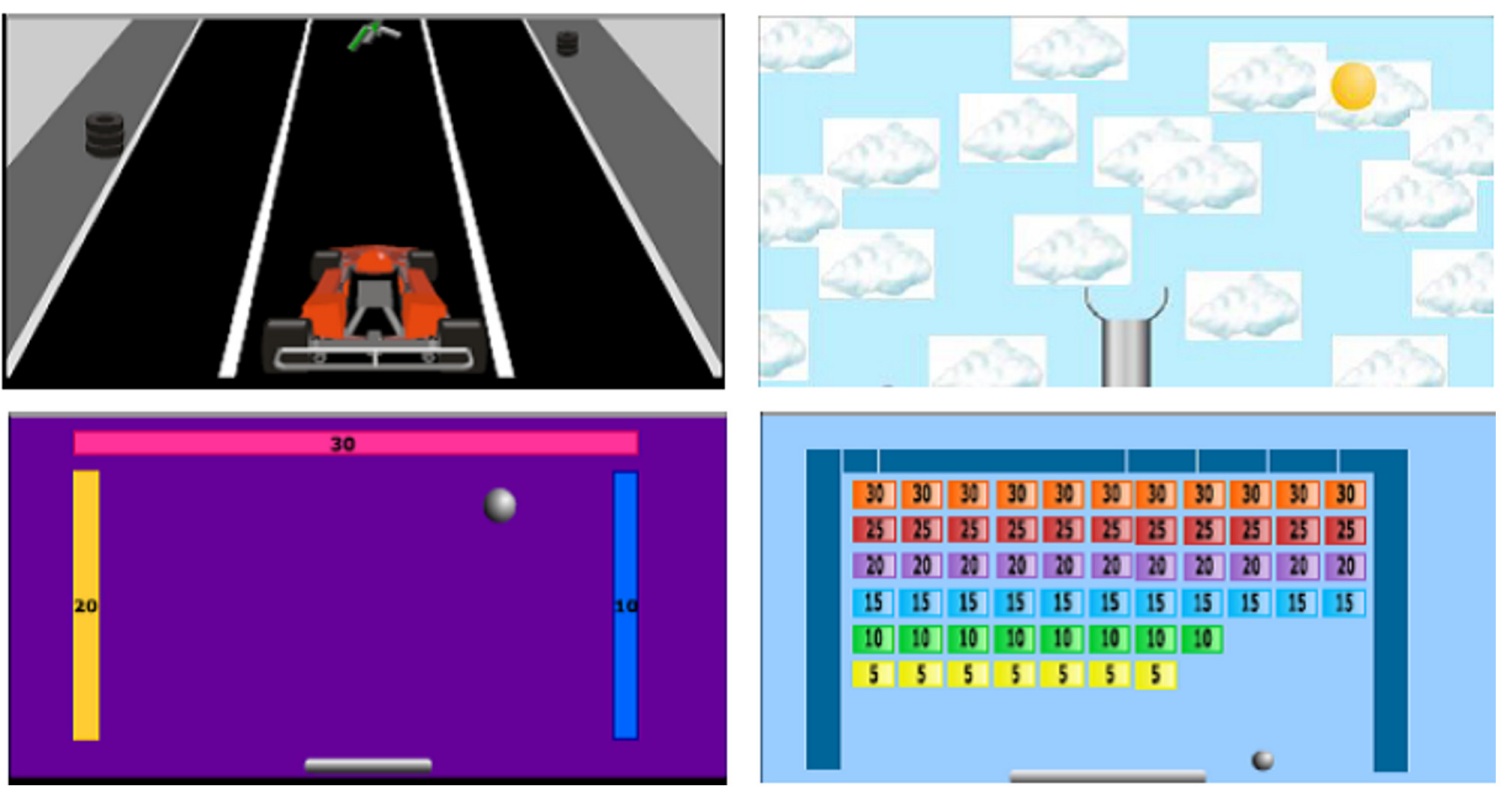
Computerised Multiple Elements Test (CMET). Each panel shows one game, which is presented to the player on a full-screen view. The games are played sequentially, with a button to press to switch to the next game.

We developed a task meeting the above requirements, called the Computerised Multiple Elements Test (CMET) [16]. The CMET comprises four simple games (see Fig 1). Each game is presented on screen one at a time, in a fixed order. Participants are instructed that they should try to score as many points as they can on each game, but that their main goal is to play each of the games a specified number of times, dividing their time fairly equally, before the overall time limit expires. Pilot work with patients with acquired brain injury indicated that the optimum overall time limit was five minutes, with instructions to play each of the four games twice, in order to minimise floor and ceiling effects in performance.

In the present paper we describe preliminary research towards validating the CMET. Our aims were to investigate if the CMET was sensitive to goal neglect in a clinical sample, and if CMET performance in healthy adults activated rostral PFC in a manner consistent with previous studies which have used more structured prospective memory paradigms.

## Study 1 Methods and Results

We hypothesised that CMET performance would correlate significantly with performance on established tests of goal neglect, in a sample of patients with acquired brain injury.

### Study 1 Participants

Patients with acquired brain injury were recruited from a regional in-patient rehabilitation unit. Inclusion criteria were: presence of acquired non-progressive brain injury; medically stable; capacity to consent to research. Exclusion criteria were: sensory or motor impairment that would preclude administration of the study tasks; memory or language impairment that would invalidate the task instructions; major psychiatric or other neurological comorbidity. Although all participants had an acquired brain injury, no further criteria were applied regarding type of injury, site of injury or presence/absence of goal neglect on clinical assessment; this was to allow for a range of performance levels on the CMET across the sample, with minimal floor and ceiling effects and a varied score distribution for the correlational analyses.

### Study 1 Materials and Procedure

The CMET was administered on a laptop computer. Participants were instructed to play each of the four games twice within a five minute period, with the aim of scoring as many points as possible; the countdown timer could be viewed by pressing a button. The primary outcome measure was performance on the CMET as measured by number of games played (maximum = 8). In addition to the CMET, two established table-top tests of goal neglect were administered: the Hotel Test and the Modified Six Elements Test from the BADS. Performance on the Hotel Test was measured by number of tasks and events completed (maximum = 7). Performance on the BADS Modified Six Elements Test was expressed as a standardised profile score (maximum = 4), derived from the BADS test manual. The three goal neglect tasks (CMET, Hotel Test and BADS Modified Six Elements Test) were always administered at the start of the testing session, with administration order within those three tasks counterbalanced across the sample. Participants were then assessed using the Wechsler Test of Adult Reading (WTAR) [17] to estimate premorbid intellect, and the Similarities and Matrix Reasoning subtests from the Wechsler Adult Intelligence Scale (WAIS-III) [18] to assess current verbal and non-verbal reasoning. The study was approved by the South Glasgow and Clyde Local Research Ethics Committee and all participants gave written informed consent.

### Study 1 Data Analysis

Correlation coefficients with 95% confidence interval (CI) were computed between the CMET score and the Hotel Test and BADS Modified Six Elements Test. Associations were also tested between the CMET score and other demographic and clinical variables (age, gender, months since injury, WTAR-predicted IQ, WAIS-III Similarities and Matrix Reasoning scale scores), using correlation coefficients or *t*-tests as appropriate. Alpha was set at p = 0.05. Analyses were conducted in SPSS v19 (Armonk, NY: IBM Corp) or Stata v13 (College Station, TX: StataCorp LP).

### Study 1 Results

Eighteen patients took part, of whom 14 (77.8%) were male. Mean age was 34.1 years (SD = 10.5). Mean WTAR-estimated pre-morbid IQ was 102.8 (SD = 9.6). Diagnoses were as follows: infarct (7; 38.9%), intra-cerebral haemorrhage (4; 22.2%), traumatic brain injury (3; 16.7%), subarachnoid haemorrhage (2; 11.1%), tumour (1; 5.6%) and hypoxic brain injury (1; 5.6%). Further information about type of injury is provided in S1 Appendix.

**Fig 2:**
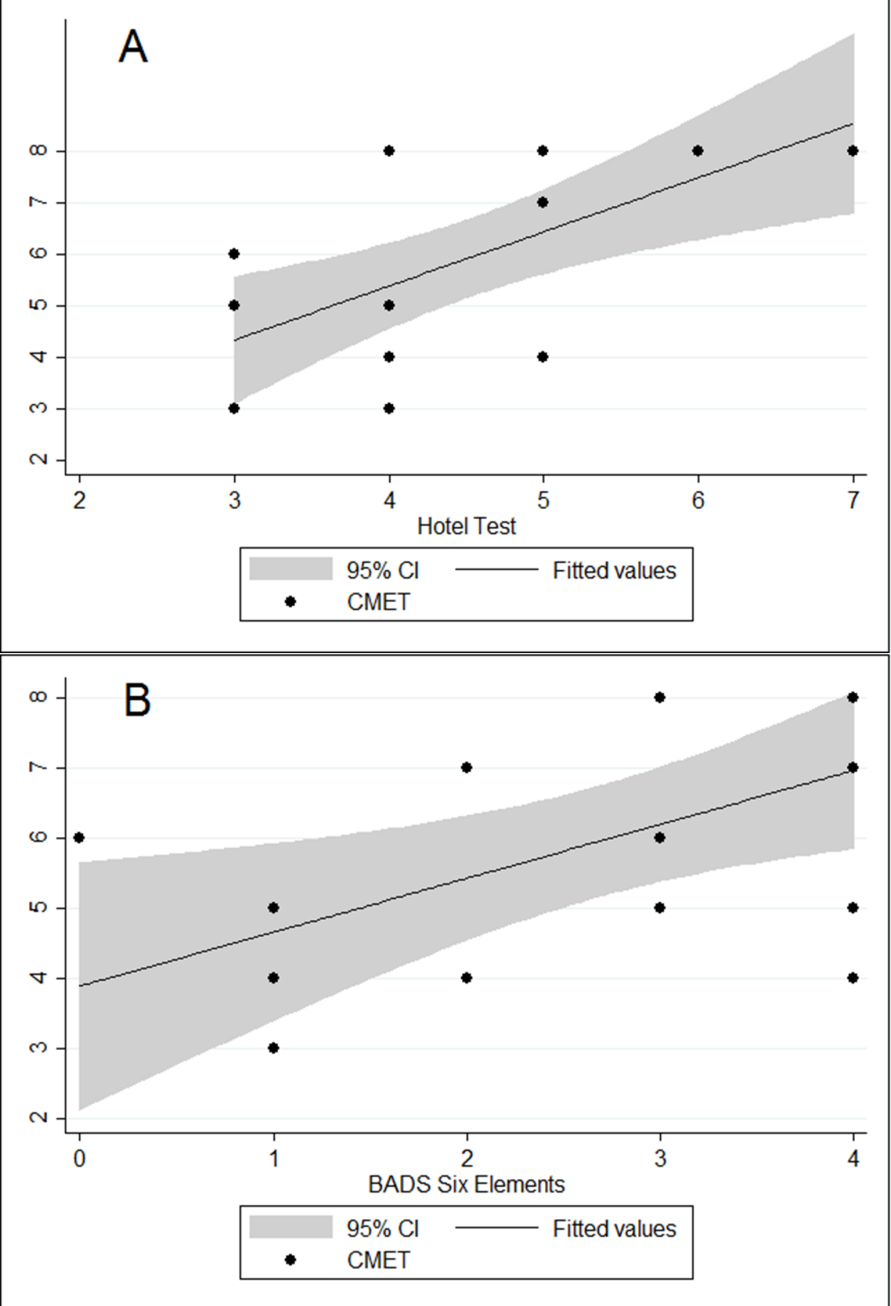
Association Between CMET Performance and Table-top Multiple Elements Tests. Panel A shows association between CMET and Hotel Test (*r* = 0.675; 95% CI 0.288, 0.872; p = 0.003; n = 17). Panel B shows association between CMET and BADS Modified Six Elements Test (*r* = 0.568; 95% CI 0.138, 0.817; p = 0.014; n = 18).

A wide range of scores was observed on the CMET, Hotel Test and Modified Six Elements Test, with no floor or ceiling effects evident. Table 1 shows the associations between CMET performance and other demographic and clinical variables. Significant positive correlations were observed between the CMET and the Hotel Test (*r* = 0.675; 95% CI 0.288, 0.872; p = 0.003; n = 17) and between the CMET and the BADS Modified Six Elements Test (*r* = 0.568; 95% CI 0.138, 0.817; p = 0.014; n = 18) (see Fig 2). A more modest correlation was observed between the Hotel Test and the BADS Modified Six Elements Test (*r* = 0.394; 95% CI -0.106, 0.735; p = 0.118; n = 17). CMET performance was not significantly associated with age, gender, months since injury, premorbid IQ, or WAIS-III subtests.

**Table 1:** Demographic and Clinical Variables and Their Association With CMET Performance (Study 1).

	Sample characteristics n = 18	Association with CMET performance
**Age (years),** M (SD)	34.1 (10.5)	*r* = -0.372, p = 0.128
**Male gender,** n (%)	14 (77.8)	*t* (16) = -0.665, p = 0.515
**Months since injury,** median (25^th^, 75^th^ percentile)	3.6 (2.5, 5.3)	rho = -0.189, p = 0.452
**WTAR-estimated IQ,** M (SD)	102.8 (9.6)	*r* = -0.102, p = 0.687
**WAIS-III Similarities,** M (SD)	7.8 (2.4)	*r* = 0.038, p = 0.882
**WAIS-III Matrix Reasoning,** M (SD)^a^	7.0 (2.8)	*r* = 0.025, p = 0.923
**Hotel Test,** M (SD)^b^	4.5 (1.2)	*r* = 0.675, p = 0.003
**BADS Modified Six Elements Test,** M (SD)	2.7 (1.4)	*r* = 0.568, p = 0.014
**CMET,** M (SD)	5.9 (1.9)	-

BADS, Behavioural Assessment of the Dysexecutive Syndrome; CMET, Computerised Multiple Elements Test; IQ, intelligence quotient; M, mean; SD, standard deviation; WAIS-III, Wechsler Adult Intelligence Scale 3^rd^ Edition; WTAR, Wechsler Test of Adult Reading.

^a^n = 17; one participant declined to complete the task.

^b^n = 17; one participant was unable to complete the task due to hemiparesis.

## Study 2 Methods and Results

We hypothesised that during CMET performance, fMRI would show increased activation in rostral PFC prior to switching between games. We predicted that rostral PFC activation would be elicited during self-directed CMET performance (which required the participant to maintain the goal intention and decide when to switch), but not during a control condition in which the researcher prompted the participant to switch (thus removing goal management demands).

### Study 2 Participants

Healthy adults (right-handed) with no history of brain injury or neurological disorder were recruited via poster advertisement in public areas at a regional hospital and via a feature in the newsletter of a local brain injury charity.

### Study 2 Materials

Participants were screened prior to scanning using the following measures: WTAR, to estimate premorbid intellectual level; BADS Modified Six Elements Test, to assess goal management performance; Cognitive Failures Questionnaire (CFQ) [19], Prospective and Retrospective Memory Questionnaire (PRMQ) [20], and BADS DEX questionnaire, to assess self-reported everyday memory failures; and Hospital Anxiety and Depression Scale (HADS) [21], to measure self-reported mood state. The CMET task was administered in the MRI scanner; participants were required to play each of the four games twice within each five minute playing period, with the countdown timer visible on screen at all times. The index and middle finger of the right hand controlled left and right movement on screen, and the index finger of the left hand controlled switching between games.

### Study 2 Procedure

The study was approved by the Greater Glasgow Primary Care Division Research Ethics Committee and all participants gave written informed consent.

#### Behavioural procedure

**P**articipants were scanned for six consecutive runs lasting five minutes each. All participants played the CMET under two conditions.

Condition 1 –Self-directed: Participants were instructed to play each of the four games twice during the run, switching between the games at any time of their choosing.

Condition 2 –Prompted: Participants were instructed to play each of the four games twice during the run, switching between the games only when verbally prompted to by the researcher. Prompts were given approximately every 37 seconds, to produce equal playing time across games.

The two conditions were designed to elicit differential fMRI activation depending on the demands for internally self-generated behaviour, while keeping actual performance behaviour (playing the games and pressing the button to switch) equal across conditions. Each participant underwent both conditions three times, in alternating order. Participants were randomised (according to a computer-generated sequence which was determined after sample recruitment) to begin with either the self-directed or the prompted condition, with conditions alternated thereafter. All participants practised the task on a laptop computer immediately prior to entering the scanning environment.

#### Functional MRI procedure

Scanning was conducted at an NHS regional neurosciences centre, using a 3-Tesla GE Signa HD MRI scanner with 8-channel head coil. The experimental task was presented via a NordicNeuroLab projector headset, with responses registered via bilateral hand-held button boxes. Whole brain, 3D T1-weighted anatomical images were acquired using an IR FSPGR sequence, TR = 6.9msec, TE = 1.6msec, TI = 500msec. Functional images were acquired using a T2*-weighted EPI BOLD sequence, TR = 2000msec and TE = 30msec. Each volume comprised 28 axial slices (4.5mm thickness with no interslice gap), orientated at approximately 10° to the anterior commissure–posterior commissure plane, covering the whole brain. One hundred and fifty volumes were acquired during each five-minute scanning run, providing a temporal resolution of 2 seconds. Additional field map scans were acquired for use in image pre-processing procedures.

### Study 2 Data Analysis

Analysis was conducted using Statistical Parametric Mapping software (SPM8; http://www.fil.ion.ucl.ac.uk/spm/). Single subject analyses were first carried out, followed by a second level random effects group analysis. For each participant, volumes were realigned and unwarped using field map data, then normalised into 2mm cubic voxels using a standard EPI template based on the Montreal Neurological Institute (MNI) reference brain, and smoothed with an 8mm full-width half-maximum Gaussian kernel. A regression model was constructed, treating each five-minute run as a separate time series. Individual two-second periods immediately preceding each left-hand button press (switching between games) were coded as events within the time series; this was because the activation of interest (decision to switch) was likely to occur within this time window. Activation associated with these event times was convolved with a canonical haemodynamic response function. No other regressors were included. Contrast images from the single subject analyses were entered into a random effects analysis, with one-sample *t*-tests to test for differential activation in the self-directed versus prompted condition (self-directed > prompted) and vice versa (prompted > self-directed). Contrasts were initially thresholded at p < 0.001 uncorrected for multiple comparisons, with a minimum extent of five contiguous voxels. Small volume correction was then applied within the *a priori* region of interest (BA10), thresholded at p < 0.05 with family-wise error correction. BA10 was defined by a mask created using the WFU PickAtlas tool v3.0 (http://fmri.wfubmc.edu/software/PickAtlas); the source was the ‘TD brodmann areas+’ atlas, from the TD-ICBM Human Atlas. The purpose of applying small volume correction in this region was to avoid Type II error that can result from stringent correction across the whole brain. Activations outside this region of interest (at the p < 0.001 uncorrected level) should be interpreted with caution. Software tools (WFU PickAtlas; XJView http://www.alivelearn.net/xjview8/) were used to map activation coordinates to anatomical regions.

### Study 2 Results

Of the 12 participants, eight (66.7%) were male and mean age was 34.6 years (SD = 7.6). Mean WTAR-estimated IQ was 111 (SD = 3.9) and mean years of education was 16.9 (SD = 3.7). All participants showed unimpaired performance on the BADS Modified Six Elements Test, thus confirming normal goal management ability. Self-report responses on the cognitive and mood questionnaires highlighted no clinical problems with everyday cognitive performance or mood state. As expected, all participants performed at ceiling on the CMET task during scanning, indicating absence of goal management problems. The structural MRI scans were reviewed by a Consultant Neuroradiologist, and one participant was found to have an incidental pineal cyst of no clinical significance.

#### Effect of self-directed condition over prompted condition

**Fig 3:**
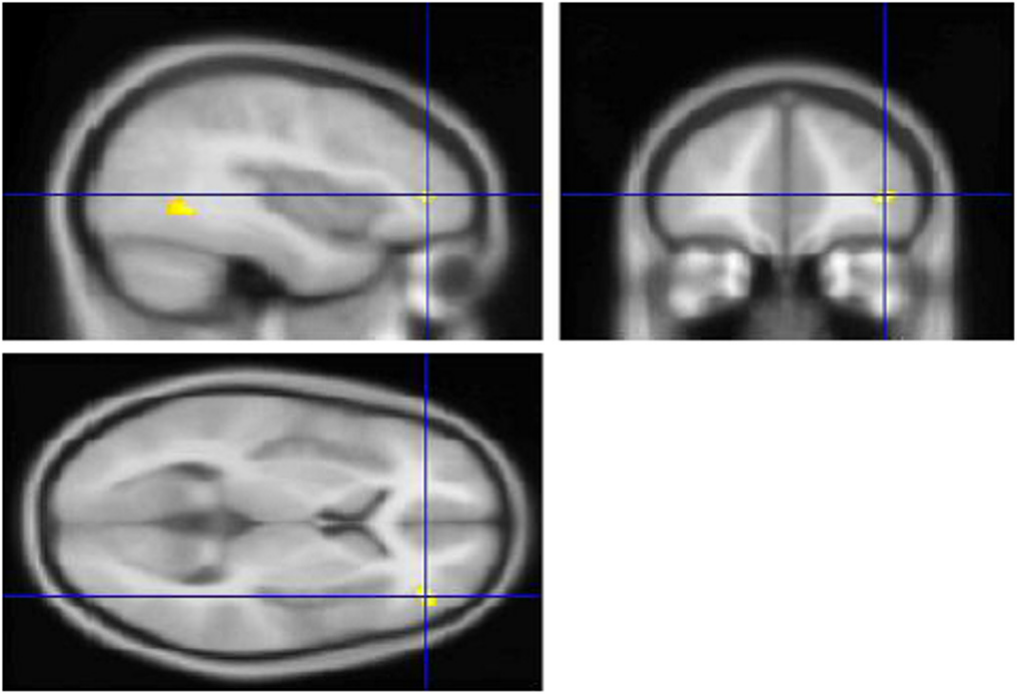
Significant fMRI Activation Cluster in Rostrolateral Prefrontal Cortex During Self-directed Condition (Self-directed > Prompted) (Study 2). Peak Montreal Neurological Institute coordinate 40, 44, 4; *Z*_E_ = 4.25; p = 0.026 (family-wise error-corrected within Brodmann Area 10).

Uncorrected (p < 0.001) results for the self-directed > prompted contrast are given in S1 Table. When family-wise error correction was applied within BA10, a significant cluster of activation was seen in right lateral cortex (MNI peak 40, 44, 4; *Z*_E_ = 4.25; p = 0.026; see Fig 3).

#### Effect of prompted condition over self-directed condition

Uncorrected (p < 0.001) results for the prompted > self-directed contrast are reported in S1 Table. When family-wise error correction was applied within BA10, no voxels survived thresholding at p < 0.05.

## Discussion

These preliminary studies provide initial evidence towards the validation of the CMET as a test of goal neglect, suitable for use in functional neuroimaging paradigms. This pilot work provides encouraging justification for further experimental research to confirm these findings and investigate other psychometric properties of the task. Study 1 demonstrated concurrent validity with established tests of goal neglect in a small clinical sample, as evidenced by significant correlation coefficients with large effect size. Discriminant validity was also indicated by the absence of statistical associations with other neuropsychological constructs measured (estimated premorbid IQ; abstract reasoning), and CMET performance did not appear to be associated with demographic variables. The results of Study 2 were congruent with the existing literature on the role of rostral PFC in maintaining and executing intentions, and provide a novel extension to this literature by demonstrating such activation on a relatively unstructured multi-element task, rather than a classic prospective memory paradigm centred on responses to targets.

The finding in Study 2 that self-directed switching on the CMET activated rostrolateral PFC is in keeping with previous functional neuroimaging studies which have linked rostrolateral PFC with maintenance of internally-mediated thought [22], switching between stimulus-oriented and stimulus-independent thought [23,24], and executing self-initiated delayed intentions [25]. Furthermore, one lesion-based study reported a significant negative correlation between volume of damage in right lateral BA10 and performance on the Hotel Test [26]. Our findings therefore add to the growing body of evidence that rostrolateral PFC has a specialised role in the type of internally-generated intention management activity that is likely impaired in patients with goal neglect, as well as indicating the ability of the CMET to elicit such activity. It must be emphasised, however, that these results are preliminary and require replication. In particular, although the uncorrected (p < 0.001) results may be taken as a basis for formulating hypotheses for future studies, by themselves they provide weak evidence for the validity of the CMET paradigm.

The CMET is brief to administer, with minimal instructions and an interface that is simple for patients to comprehend and use. It fulfils many of Burgess’s characteristics of a multi-tasking situation [27]: it contains several tasks which must be completed one at a time; it requires acting on delayed intentions; performance (decision to switch) is self-determined; and the player receives no immediate feedback. It does not have the complexity of other computerised executive function tasks such as the JEF [28] or the Virtual MET [29], but the design and analysis constraints of functional neuroimaging paradigms render such complex tasks unsuitable, and so simpler tasks are required for this purpose.

We acknowledge that our clinical sample in Study 1 was small, and we do not propose that the CMET is ready for use as a clinical assessment at this point. Further validation studies are required to confirm the present findings, to determine classification accuracy in detecting goal neglect, and to determine the test-retest reliability of the CMET and its sensitivity to behavioural change following cognitive rehabilitation. It would also be of interest to investigate in a larger clinical sample whether poorer CMET performance is associated with anterior prefrontal damage specifically. A limitation of our study is the absence of information regarding lesion location or extent in the participants with acquired brain injury. As indicated in S1 Appendix, the sample was heterogeneous with regard to injury type; while this allowed us to obtain a range of performance levels on the behavioural measures, such heterogeneity in a small sample precludes meaningful analysis of the correspondence between goal management performance and structural pathology.

Our aim is that the CMET will in future be used to elicit goal management-related neural activation in functional neuroimaging studies of patients with brain injury, before and after rehabilitation. Although the present studies are an encouraging first step towards that goal, there remain important methodological barriers to conducting functional neuroimaging research in neurological populations [30]. Standard spatial normalisation techniques may not be successful in the presence of focal or diffuse structural lesions, and assumptions about the haemodynamic response function may not hold true. Nevertheless, important methodological research in recent years has yielded new ways to overcome these technical challenges [31], underlining the continued importance of striving to develop new experimental tasks to answer the research questions that current and future technical advances will allow us to ask. There are always challenges, however, in interpreting patterns of neural activation when patients do not perform normally on experimental tasks compared to controls [32]: differences in activation may be a proxy for task difficulty rather than specific task requirements. In Study 2, healthy participants performed at ceiling on the CMET, whereas some performance variation in the normal population is generally desirable on any scanning task. We will therefore aim in future studies to manipulate the demands of the CMET with the aim of reducing this ceiling effect, and explore the sensitivity of other task-related measures beyond number of games completed (e.g. deviation from optimum switching time).

Differential task performance between patients and controls can also cause problems in applying event-based analysis approaches, which are the mainstay of functional imaging research in neuropsychology. These study designs assume the presence of discrete events at known times, yet the CMET (or indeed any other task designed to capture the relatively unstructured nature of goal management behaviour) leaves the decision to switch games (the event) to the participant, and those participants who show goal neglect may not switch often enough to allow event-related analysis to be applied. Future functional imaging research using the CMET may require a different approach, e.g. model-free techniques such as independent components analysis, which are more commonly employed in resting state paradigms.

## Supporting Information

S1 Appendix Brain injury sub-types in participants of Study 1 (n = 18). (DOCX)

S1 Table fMRI activation clusters in each condition (Study 2). (DOCX)
