# Supplementary material for: Towards Validation of a New Computerised Test of Goal Neglect: Preliminary Evidence from Clinical and Neuroimaging Pilot Studies
Source: PLoS One. 2016 Jan 29;11(1):e0148127. doi: 10.1371/journal.pone.0148127 (PMC4732681; doi:10.1371/journal.pone.0148127)
Supplement: S1 Appendix — (DOCX) [file pone.0148127.s001.docx]

**S1 Appendix. Brain injury sub-types in participants of Study 1 (n = 18).**

Infarct (n = 7; 38.9%)

- Right middle cerebral artery infarct (n = 3)
- Large right temporo-parietal and basal ganglia infarct
- Right hemisphere infarct
- Left anterior cerebral artery infarct
- Multiple embolic infarcts

Intracerebral haemorrhage (n = 4; 22.2%)

- Left intracerebral haematoma
- Right intracerebral haematoma
- Basal ganglia haemorrhage
- Ruptured right frontal arteriovenous malformation

Traumatic brain injury (TBI) (n = 3; 16.7%)

- TBI with chronic subdural haematoma
- TBI fall
- TBI struck by car

Subarachnoid haemorrhage (n = 2; 11.1%)

- Ruptured right middle cerebral artery aneurysm
- Ruptured right anterior choroidal artery aneurysm

Tumour (n = 1; 5.6%)

- Pituitary adenoma

Hypoxic brain injury (n = 1; 5.6%)

- Hypoxia during status epilepticus
