# Supplementary material for: Towards Validation of a New Computerised Test of Goal Neglect: Preliminary Evidence from Clinical and Neuroimaging Pilot Studies
Source: PLoS One. 2016 Jan 29;11(1):e0148127. doi: 10.1371/journal.pone.0148127 (PMC4732681; doi:10.1371/journal.pone.0148127)
Supplement: S1 Table — (DOCX) [file pone.0148127.s002.docx]

**S1 Table. fMRI activation clusters in each condition (Study 2).**

| **Region** | **BA** | **Hemisphere** | **x** | **y** | **z** | ***Z*_E_** | ***N* voxels** |
| --- | --- | --- | --- | --- | --- | --- | --- |
| *Self-directed > prompted* | | | | | | | |
| Rostrolateral PFC | 10/46 | R | 40 | 44 | 4 | 4.25* | 33 |
| Inferior parietal lobe (white matter) | - | R | 26 | -54 | 34 | 3.65 | 133 |
| Posterior temporal lobe (white matter) | -  - | R  R | 40  30 | -52  -46 | -4  14 | 3.62  3.66 | 59  22 |
| Limbic lobe | - | L | -36 | -42 | -8 | 3.27 | 6 |
| Cerebellar tonsil | - | L | -22 | -42 | -48 | 3.25 | 9 |
| *Prompted > self-directed* | | | | | | | |
| Rostromedial PFC | 9/10 | L | -2 | 60 | 30 | 3.65 | 42 |
| Inferior frontal gyrus | 47 | R | 46 | 32 | -8 | 4.06 | 116 |
| Superior/middle temporal gyri | 21/22/41  21/22/41 | R  L | 56  -62 | -8  -44 | -16  12 | 6.03  5.57 | 3733  3733 |
| Angular gyrus | 39  39 | R  L | 54  -46 | -64  -74 | 30  30 | 4.14  3.81 | 83  64 |
| Precuneus | 7/31 | R | 6 | -66 | 32 | 3.94 | 828 |
| Limbic lobe | - | R | 24 | -2 | -22 | 3.90 | 84 |
| Posterior cerebellum | - | L | -22 | -78 | -44 | 3.28 | 9 |

BA, Brodmann area; L, left; PFC, prefrontal cortex; R, right. All activations significant at p < 0.001 uncorrected for multiple comparisons, and * = significant at p < 0.05 with family-wise error correction within BA10. Activations significant at p < 0.001 uncorrected should be interpreted with caution. Coordinates refer to the Montreal Neurological Institute reference brain. Brodmann areas are approximate.
